# HHLA2: a potential biomarker and therapeutic target in endocrine-related cancer

**DOI:** 10.1530/EO-24-0034

**Published:** 2025-04-07

**Authors:** Christiane Gruetzmacher, Bruna Sousa Pessoa, Flora Ladeira Craveiro, Marilena Nakaguma, Ericka Barbosa Trarbach, Rafael Loch Batista

**Affiliations:** ^1^ Neuroendocrinology Unit, Division of Endocrinology and Metabolism, Hospital das Clínicas, Faculty of Medicine, University of São Paulo, São Paulo, Brazil; ^2^ Laboratorio de Endocrinologia Celular e Molecular, LIM25, Disciplina de Endocrinologia e Metabologia, Hospital das Clinicas HCFMUSP, Faculdade de Medicina, Universidade de São Paulo, São Paulo, SP, Brazil; ^3^ Division of Endocrine Oncology, Cancer Institute of the State of São Paulo (ICESP), Faculty of Medicine, University of São Paulo, São Paulo, Brazil; ^4^ Laboratory of Medical Investigations 42 (LIM42), Medicine School, University of São Paulo, São Paulo, Brazil

**Keywords:** HHLA2, biomarker, therapeutic target, endocrine-related cancer, immune checkpoint, cancer immunotherapy

## Abstract

**Purpose:**

Human endogenous retrovirus-H long terminal repeat-associating 2 (HHLA2), a member of the B7 family, is widely expressed across human cancers and is emerging as a promising immune checkpoint target for therapeutic development. This study aims to consolidate existing data on HHLA2 expression in endocrine-related cancers and evaluate its potential as a prognostic biomarker.

**Methods:**

Original studies published in English up to December 2024 were searched using PubMed, Web of Science and Embase databases. Search strategies combined MeSH terms and keywords related to ‘HHLA2’, ‘B7-H7’, ‘B7y’, ‘B7-H5’ and ‘cancer’, with a specific focus on endocrine-related cancers.

**Results:**

From a total of 117 studies reviewed, twelve met the inclusion criteria. Seven studies on pancreatic cancer indicated varied HHLA2 expression patterns, with high expression levels associated with better prognosis and improved overall survival. In ovarian cancer, one study suggested that high HHLA2 expression in tumor cells could predict improved survival. In contrast, another study linked HHLA2 to lymph node metastasis and poor overall survival, observing high expression only in stromal cells. On the other hand, studies on thyroid cancer and neuroendocrine tumors highlighted HHLA2’s significance in disease progression, indicating poor prognosis and its association with metastasis.

**Conclusion:**

HHLA2 plays dual roles, exhibiting both immunosuppressive and tumor-suppressive functions in endocrine-related tumors, with its expression possibly influenced by the tumor microenvironment. This highlights its promise as an immune checkpoint biomarker and therapeutic target. The collective data from this review provide insights for future research endeavors in HHLA2-associated oncology.

## Introduction

Human endogenous retrovirus-H long terminal repeat (LTR)-associating 2 (HHLA2), also known as B7-H5, B7-H7 or B7y, was initially recognized for its polyadenylated LTR sequence derived from human endogenous retrovirus-H (HERV-H) in its 3′ untranslated region (Zhao *et al.* 2013, Ying *et al.* 2022). HERV-H, along with other human endogenous retroviruses, represent remnants of ancient retroviral DNA insertions that have integrated into the human germline throughout evolution, significantly influencing the innate immune system (Zhang *et al.* 2019). Furthermore, phylogenetic analysis has confirmed that HHLA2 is a member of the B7/CD28 family, a critical group that modulates immune responses via interactions with CD28 receptors on lymphocytes (Zhao *et al.* 2013, Ying *et al.* 2022). Members of this family are characterized by having both variable (V) and constant (C) type domains of the immunoglobulin superfamily (Zhao *et al.* 2013).

The tumor microenvironment (TME), consisting of various immune and non-immune cells and the extracellular matrix, plays a critical role in tumor behavior. Interestingly, HHLA2 interacts with two main receptors: transmembrane and immunoglobulin domain-containing 2 (TMIGD2) and killer cell immunoglobulin-like receptor, three Ig domains and long cytoplasmic tail 3 (KIR3DL3). Through these interactions, HHLA2 can deliver either stimulatory or inhibitory signals, respectively, thereby affecting the immune system’s ability to detect and target cancer cells (Ying *et al.* 2022).

HHLA2 expression is elevated in a variety of tumors compared to adjacent non-tumoral or healthy tissues (Ying *et al.* 2022, Mortezaee 2023), which suggests a significant role for this protein in the processes of tumorigenesis and cancer progression. Notably, high levels of the HHLA2 protein correlate with increased tumor size and stage, lymph node metastasis and lower rates of relapse-free and overall survival in affected patients (Kula *et al.* 2024). These associations suggest that HHLA2 could serve as a promising prognostic biomarker. Beyond its potential as a biomarker, HHLA2 also presents itself as a target for immunotherapy, especially in patients who are resistant to other immune checkpoint inhibitors (ICIs) such as programmed cell death 1 (PD-1) and cytotoxic T-lymphocyte antigen-4 (CTLA-4) inhibitors (Stricker *et al.* 2023). Interestingly, HHLA2 is often expressed more frequently than the PD-1 ligand (PD-L1) and does not overlap with PD-L1 expression in certain cancers, distinguishing the KIR3DL3/TMIGD2-HHLA2 pathway as an innovative avenue for cancer immunotherapy (Bhatt *et al.* 2021, Ying *et al.* 2022). Noteworthily, HHLA2 has limited expression in healthy tissues, predominantly found in organs requiring immune regulation, such as the gastrointestinal system and placenta (Janakiram *et al.* 2017).

ICIs are increasingly recognized as potential immunotherapeutic targets in various human cancers and have transformed cancer treatment by enhancing immune responses against tumors (Niu *et al.* 2022*a*, Ying *et al.* 2022). By stimulating the host’s immune system and blocking inhibitory signals, ICIs have shown promising results in treating various types of cancer (Ying *et al.* 2022). However, a significant number of patients with advanced-stage cancers still exhibit primary or secondary resistance to ICIs, which may be due to *de novo* or adaptive resistance (Zhou *et al.* 2022). Furthermore, some patients may experience severe side effects, including toxicity and tumor hyper-progression. Therefore, it is critical to identify alternative immune checkpoint pathways and develop novel cancer-fighting strategies, including combination therapies that target multiple checkpoints (Park *et al.* 2021, Kamali *et al.* 2023).

Exploring HHLA2 in the field of oncology has emerged as a promising research area, although its full potential remains largely unexplored. In this review, we aim to provide a comprehensive analysis of the diverse roles of HHLA2 in oncology, focusing particularly on its implications in endocrine-related cancers. Our goal is to elucidate its clinical significance and proposed functions in tumor biology.

## Materials and methods

A rigorous literature analysis was performed to explore the relationship between HHLA2 expression and endocrine-related cancers, as well as to assess its implications for the prognostic outcomes of these tumors. PubMed, EMBASE and Web of Science databases were searched using the terms ‘HHLA2’ OR ‘B7-H7’ OR ‘B7y’ OR ‘B7-H5’ AND ‘endocrine-related cancers’ OR ‘endocrine cancers’ OR ‘thyroid cancer’ OR ‘ovarian cancer’ OR ‘pancreatic cancer’ OR ‘neuroendocrine tumors’ OR ‘adrenocortical cancer’ OR ‘testicular germ cell tumor’ OR ‘pheochromocytoma/paraganglioma’, covering the period until December 2024.

To enhance the sensitivity of the search strategy, disease-related keywords such as ‘endocrine-related cancers’ OR ‘endocrine cancers’ were entered as free-text terms, which generated a substantial number of results. An extensive search for outcomes was also performed, including metrics such as overall survival, prognosis, survival, biomarker status, metastasis, disease-free survival, progression-free survival, tumor mutation burden and microsatellite instability. This was supplemented by a manual review of the citation lists from selected studies to identify any additional publications that might have been missed in the electronic search. Only studies published in English were included in this analysis.

## Results

### Study selection and characteristics of the included studies

**Figure 1:**
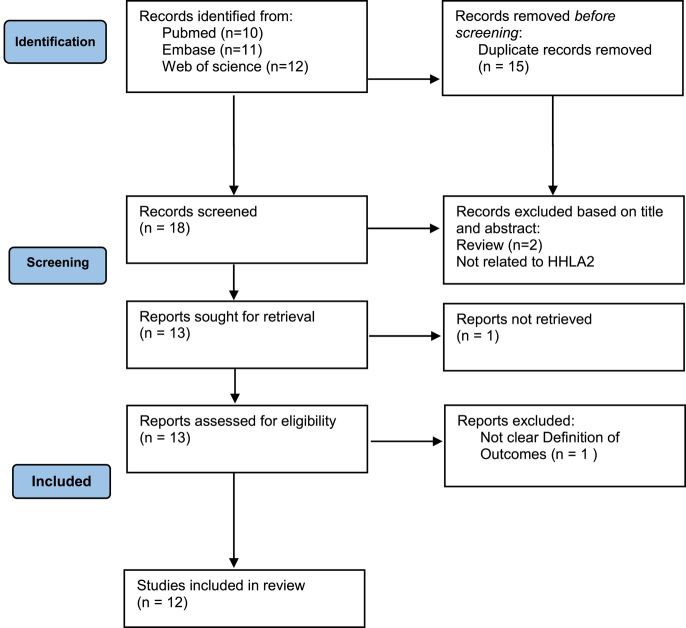
Flow chart of included studies.

Through a systematic database search, 34 articles were initially retrieved. After removing 15 duplicates, the titles and abstracts of the remaining articles were screened, leaving 13 articles for full-text review. During this process, one publication was unavailable, two studies were classified as reviews, three did not relate to HHLA2 expression, and one lacked a clearly defined outcome, leading to their exclusion. An additional study was identified through the reference lists of the selected studies, as shown in Fig. 1. Finally, 12 studies were included in the final review.

These studies specifically examined HHLA2 expression across a range of endocrine cancer types: five studies focused on pancreatic cancer (PC), two on ovarian cancer (OC), and one study each on papillary thyroid carcinoma (PTC), medullary thyroid carcinoma (MTC) and gastrointestinal neuroendocrine tumors (GINETs) along with pancreatic neuroendocrine tumors (PNETs). In addition, one study assessed HHLA2 expression in PC using bioinformatic methods from database analyses (Table 1).

**Table 1:** HHLA2 expression and its association with prognosis and outcomes in endocrine-related cancers.

Author (year)	Cancer type	Sample size	Detection method	IHC scoring/cutoff*	Prognostic implications of HHLA2 expression
Byers *et al.* (2015)	PC	23	IHC	Staining intensity/-	None
Chen *et al.* (2019)	PC	136	IHC	Percentage of positive cells/>25%	Longer OS for high expression
Yan *et al.* (2019)	PC	92	IHC	Percentage of positive cells/>6%	Better prognosis for high expression
Boor *et al.* (2020)	PC	122	IHC	Staining intensity/Intermediate or stronger	Improved post-surgical survival for high expression
Zhu *et al.* (2022)	PC	63	mIHC	Percentage of positive cells/>26.7%**	High expression in TAM was associated with a poor prognosis; high expression in tumor cells tended to better OS
Huang *et al.* (2023)	PC	179	RNA seq	-	High mRNA levels was associated with shorter OS
Aydın & Turhan (2024)	PC	92	IHC	Percentage of positive cells/>50%	Low expression was associated with high perineural invasion and with high pathological stage
Fu *et al.* (2020)	OC	119	mIHC	Percentage of positive cells/>31.51%**	High expression in stroma was linked to advanced stages and poor prognosis
Xu *et al.* (2021*a*)	OC	64	IHC	Percentage of positive cells/>0%	High expression was associated with well differentiated cells, high CD8+ lymphocytes levels and survival
Niu *et al.* (2022*a*)	PTC	107	qPCR	-	High mRNA levels suggest advanced cancer stages and poor survival
Niu *et al.* (2022*b*)	MTC	51	IHC	H score/>2	High expression was associated with lymph node metastasis and poor survival rates
Yuan *et al.* (2021)	GINET/ PNETs	13/24	IHC/qPCR	Percentage of positive cells/-	High expression was correlated with higher nodal/distant spread, tumor size and proliferation

* Cutoff for high expression of HHLA2 protein evaluated by IHC.

** Median score was used to determine the cutoff point between high or low expression of HHLA2 protein.

PC, pancreatic cancer; OC, ovarian cancer; PTC, papillary thyroid carcinoma; MTC, medullary thyroid carcinoma; GINETs, gastrointestinal neuroendocrine tumors; PNETs, pancreatic neuroendocrine tumors; IHC, immunohistochemistry; mIHC, multiplex IHC; qPCR, quantitative polymerase chain reaction.

### Understanding the immunoregulatory role of HHLA2

HHLA2 exerts a pivotal role in orchestrating the regulation of the immune system, concurrently affecting T cell proliferation, function and cytokine production through its two receptors, TMIGD2 and the recently identified KIR3DL3 (Ying *et al.* 2022, Li *et al.* 2023). TMIGD2 is an immunostimulatory receptor predominantly expressed on naïve T cells and natural killer (NK) cells. It plays a crucial role in delivering co-stimulatory signals that enhance T cell growth and cytokine production, such as IL-17, IL-5, IL-10, IFN-γ and TNF-α, via an AKT-dependent signaling cascade (Kula *et al.* 2024). TMIGD2 expression has been found to be significantly upregulated in glioma patients, correlating with better overall survival and increased immune cell infiltration, and is negatively associated with pathways such as angiogenesis and hypoxia, suggesting its role in inhibiting tumor progression (Boulhen *et al.* 2023).

**Figure 2:**
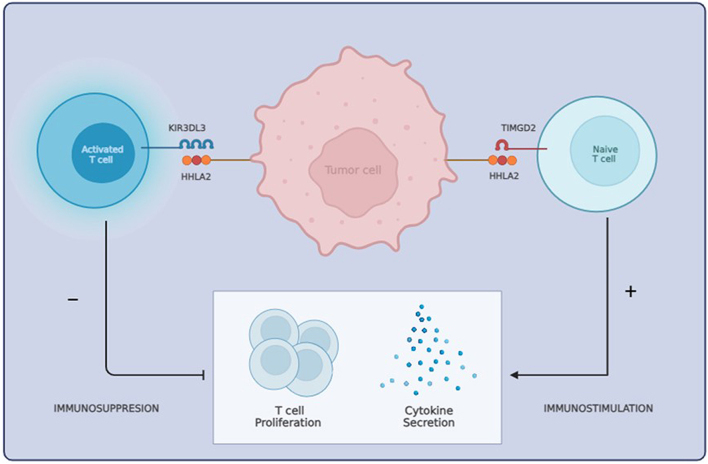
The dual role of HHLA2 within the TME. HHLA2 modulates immune responses via its co-stimulatory receptor, TMIGD2 and co-inhibitory receptor, KIR3DL3, expressed on naïve and activated T/NK cells. These receptors engage with HHLA2 in distinct spatial and temporal dynamics. HHLA2’s interaction with TMIGD2 on naïve T cells and primary NK cells enhances tumor killing by boosting T cell proliferation, cytokine secretion and the lysing functions of both NK cells and T cells. Conversely, as T/NK cells activate, TMIGD2 expression decreases while KIR3DL3 expression increases. The binding of HHLA2 to KIR3DL3 on activated T/NK cells inhibits T cell proliferation and cytokine production, highlighting its dual regulatory impact in the dynamic TME.

In contrast, as T/NK cells undergo activation, a dynamic shift occurs: TMIGD2 expression is downregulated, whereas KIR3DL3 expression is upregulated (Wei *et al.* 2021). The binding of HHLA2 to KIR3DL3 on activated T/NK cells prompts a co-inhibitory effect, targeting T cell proliferation and cytokine production. KIR3DL3 recruits SHP-1 and SHP-2 phosphatases, which attenuate signaling pathways such as ERK1/2, AKT and NF-κB, leading to reduced immune cell activation and function, thereby facilitating tumor immune evasion (Wei *et al.* 2021). HHLA2+ tumors from human kidney, lung, gallbladder and stomach were infiltrated by KIR3DL3+ immune cells. KIR3DL3 blockade has been shown to inhibit tumor growth in various humanized mouse models, suggesting its potential as an immunotherapeutic target (Wei *et al.* 2021). The dual regulatory mechanism of HHLA2 through TMIGD2 and KIR3DL3 underscores the dynamic and context-dependent nature of this within the evolving TME (Fig. 2).

### HHLA2 in PC

An initial investigation in 23 pancreas neoplasms conducted by Byers *et al.* showed that HHLA2 protein expression was present in normal ductal epithelium but absent in both acinar and islet cells (Byers *et al.* 2015). In intraductal papillary mucinous neoplasms (IPMN; *n* = 4), HHLA2 expression varied with pathological grade, showing strong staining in moderate-grade ducts, while high-grade IPMN lesions exhibited moderate staining. Adenocarcinomas (*n* = 15) demonstrated reduced or absent expression, and no expression was found in neuroendocrine (*n* = 2), pseudopapillary (*n* = 1) or gastrointestinal stromal tumor (*n* = 1) subtypes, whereas adjacent normal ducts showed high HHLA2 positivity. Although this study suggests that loss of HHLA2 may contribute to immune evasion of pancreatic adenocarcinoma, it does not provide a direct correlation with clinical prognosis or specific survival outcomes.

Building on these findings, Yan *et al.* reported that HHLA2 was present in 77.17% of pancreatic ductal adenocarcinoma (PDAC) cases and that its higher expression was correlated with better post-surgical survival rates (Yan *et al.* 2019). Chen *et al.* found that HHLA2 was strongly expressed in 68.38% of PDAC patients and was linked to longer overall survival; in this study, PDAC cells with high HHLA2 expression triggered a stronger immune response than low-expression cells when cocultured with T cells and, in immune-deficient mice, high HHLA2 expression inhibited PDAC growth post T-cell transfusion (Chen *et al.* 2019). Boor *et al.* observed that HHLA2 is expressed in 67% of PC and 93% of ampullary tumors, with high expression levels significantly associated with improved cancer-specific survival and delayed recurrence post-surgery, establishing HHLA2 as an independent prognostic marker (Boor *et al.* 2020).

Further investigation conducted by Zhu *et al.* explored HHLA2 expression in PC tissues using multiplex immunohistochemistry and found that HHLA2 expression in tumor-associated macrophages (TAMs) is linked to poorer prognosis (Zhu *et al.* 2022). However, high expression of HHLA2 in tumor cells tended to be associated with better overall survival, although this was not statistically significant (*P* = 0.08). Aydın and Turhan examined HHLA2 expression in adenocarcinomas of the pancreas, ampulla and distal bile duct. They found that high HHLA2 expression is associated with older patient age and reduced perineural invasion, though no significant correlation with other immunophenotypic markers was observed (Aydın & Turhan 2024). In the paper by Huang *et al.*, the researchers primarily focused on identifying PD-1 similarity genes encoding the immunoglobulin V-set domain in monocytes related to type 1 diabetes mellitus and PC prognosis (Huang *et al.* 2023). While the study investigated several genes, it notably found that high expression of HHLA2 mRNA, contrary to previous studies evaluating protein expression, was significantly correlated with shorter overall survival in PC patients.

Intriguingly, a correlation between HHLA2 and hepatitis A virus cellular receptor type-1 (HAVCR1) expression has been identified in PC (Liu *et al.* 2022). Within the TME, their combined expression patterns significantly impact various immune cell populations. The interaction not only affects T cell responses but also influences macrophage polarization, dendritic cell function and overall immune surveillance capabilities. This complex interplay may explain the varied immunological landscapes observed across different tumor types and could account for heterogeneous responses to current immunotherapy approaches. From a clinical perspective, the co-expression of HAVCR1 and HHLA2 correlates with specific patient outcomes and treatment responses, potentially exacerbating the progression and development of tumors, thereby impacting the clinical outcomes of these patients (Liu *et al.* 2022).

### HHLA2 in OC

Xu *et al.* focused on the expression of HHLA2 in epithelial OC tissues. Their findings revealed that HHLA2 expression was present in 17.2% of OC cases and significantly associated with better tumor differentiation and increased density of CD8+ tumor-infiltrating lymphocytes (Xu *et al.* 2021*a*). Notably, HHLA2 emerged as an independent prognostic factor, predicting improved survival outcomes for patients, suggesting its potential as a beneficial prognostic biomarker in OC (Xu *et al.* 2021*a*). Conversely, the study by Fu *et al.* examined HHLA2 expression across tumor and stromal compartments in epithelial OC (Fu *et al.* 2020). They found widespread expression of HHLA2, with a particular emphasis on its presence in the stromal compartment. High stromal HHLA2 expression correlated with poorer prognosis and lower overall survival rates, whereas its expression in the tumor compartment did not show a significant association with overall survival (Fu *et al.* 2020). This highlights the complex role of the TME in modulating cancer progression and patient outcomes.

In addition, subsequent research explored the effects of HHLA2 downregulation in OC cells, revealing that reducing HHLA2 inhibited cell proliferation, migration and invasion through the NF-κB signaling pathway, which is involved in processes such as chemoresistance, cancer stem cell sustenance, metastasis and immune evasion (Fu *et al.* 2023). This was linked to decreased expression of CA9, a critical protein for pH regulation also associated with cancer progression and metastasis. Downregulation of HHLA2 led to reduced CA9 at both mRNA and protein levels, while CA9 overexpression could reverse the inhibitory effects, further supporting HHLA2’s involvement in OC biology.

MUC16 (CA125) is a well-known tumor biomarker utilized in OC screening and is recognized for its immunosuppressive effects by interacting with the Siglec-9 receptor on NK cells, B cells and monocytes (Felder *et al.* 2019). A recent study performed a comprehensive analysis using clinical samples to establish correlations among peripheral blood cell proportions, serum inflammatory-related factors and MUC16 (CA125) levels (Felder *et al.* 2019). These findings revealed significant positive correlations between serum MUC16 levels and peripheral blood neutrophil counts, neutrophil-to-lymphocyte ratios, as well as inflammatory factors such as IL-6, IL-8, IL-10 and IL-2R. Notably, Siglec-9 was expressed on neutrophils and positively associated with neutrophil infiltration in OC. In addition, these neutrophils exhibited heightened expression of immunosuppression-related factors, including HHLA2 (Wu *et al.* 2023). This suggests the potential for HHLA2 expression to be a consequence of inflammatory activity triggered by cellular stimulation induced by a tumor product, specifically CA125 in this instance. This implies that heightened HHLA2 expression could potentially occur in tissues beyond the tumor site.

### HHLA2 in thyroid cancer

Two studies conducted by Niu *et al.* investigate the role of HHLA2 expression in thyroid cancers (Niu *et al.* 2022*a*,*b*). In the study focusing on PTC, Niu *et al.* found that HHLA2 mRNA was significantly overexpressed in cancer tissues compared to normal thyroid tissues (Niu *et al.* 2022*a*). This overexpression was associated with lymph node metastasis and advanced TNM stages, suggesting that HHLA2 plays a critical role in tumor progression. Furthermore, HHLA2 was identified as an independent prognostic factor, correlating with a poorer survival rate in PTC patients. *In vitro* experiments showed that HHLA2 promotes PTC cell progression, indicating its function as a tumor promoter, thereby suggesting that targeting HHLA2 could represent a novel therapeutic approach for PTC (Niu *et al.* 2022*a*).

On the other hand, a study on MTC revealed that HHLA2 expression was confined to tumor tissues and absent in adjacent noncancerous tissues. High HHLA2 expression was observed in 31.4% of patients with MTC and was significantly linked to lymph node metastasis and advanced cancer stages (Niu *et al.* 2022*b*). Notably, there was an inverse relationship between HHLA2 expression and CD8+ tumor-infiltrating lymphocytes, indicating a possible role in immune evasion. HHLA2 was also established as an independent prognostic factor for disease-free survival in MTC patients, underscoring its impact on tumor immunity and progression (Niu *et al.* 2022*b*). By retrieving data from multiple databases, it was found that the expression level of TMIGD2, a costimulatory receptor of HHLA2, in thyroid cancer is higher than that in normal thyroid tissue, and its expression level affects the malignancy of thyroid tumors and even increases their adverse prognosis (Janakiram *et al.* 2015, Zhang *et al.* 2023). By integrating these findings, it becomes evident that the HHLA2-TMIGD2 pathway plays a crucial role in cancer progression and immune regulation, offering prospects for future research and therapeutic development.

### HHLA2 and neuroendocrine tumors

The article by Yuan *et al.* explores the critical role of B7 immune checkpoints, particularly B7x and HHLA2, in the development and progression of GINETs and PNETs. The research demonstrates that both B7x and HHLA2, at mRNA and protein levels, are significantly overexpressed in tumor tissues compared to adjacent normal tissues (Yuan *et al.* 2021). This overexpression is strongly correlated with unfavorable clinical outcomes, such as higher tumor grade and a greater likelihood of nodal and distant metastases. One of the pivotal discoveries of the study is the link between B7x expression and immune evasion. The research shows that high levels of B7x are associated with reduced infiltration of CD8+ T cells in tumors, indicating a mechanism by which tumors might escape immune surveillance (Yuan *et al.* 2021). This is further supported by experiments in Men1 knockout mouse models, where the deletion of B7x led to increased T-cell infiltration and reduced tumor burden (Yuan *et al.* 2021). These findings suggest that B7x and HHLA2 could serve as valuable prognostic markers and potential therapeutic targets.

## Overall discussion

HHLA2, a member of the B7 family of immune checkpoint molecules, shows varied expression patterns and roles in different cancer types, influencing immune responses and patient prognosis (Ying *et al.* 2022, Li *et al.* 2023). In pancreatic cancer, the positive correlation between HHLA2 expression and improved survival in most studies suggests that it may enhance antitumor immune responses, possibly by facilitating T-cell infiltration and activation, making it a potentially favorable prognostic marker in PC (Byers *et al.* 2015, Chen *et al.* 2019, Yan *et al.* 2019, Boor *et al.* 2020, Zhang *et al.* 2021, Aydın & Turhan 2024). However, the dichotomy in HHLA2 expression between tumor cells and TAMs highlights the complexity of its role in tumor progression and immune modulation, with one study observing that HHLA2 expression in TAMs was correlated with poorer prognosis in PC (Zhu *et al.* 2022). In addition, in OC, the contrasting effects of HHLA2 expression in tumor versus stromal compartments illustrate the importance of the TME in influencing cancer outcomes (Fu *et al.* 2020). The association of stromal HHLA2 with poorer prognosis suggests that the tumor-supportive stroma can influence the overall impact of HHLA2 on cancer progression.

In thyroid cancer, particularly PTC, HHLA2’s role as a tumor promoter and its association with aggressive disease features highlight its potential as a therapeutic target (Niu *et al.* 2022*a*). In addition, in MTC, there is evidence of a relationship between HHLA2 expression, tumor progression and diminished disease-free survival (Niu *et al.* 2022*b*). The inverse relationship with immune cell infiltration in MTC further supports its role in immune evasion. Finally, in the context of GINETs and PNETs, HHLA2 overexpression has also correlated with a higher grade and an increased incidence of nodal and distant spread, suggesting a potential role for HHLA2 in contributing to tumor progression and metastasis by interactions with immune cell populations within the TME (Yuan *et al.* 2021).

Recent studies have revealed a contrast in HHLA2 expression between tumor cells and the TME in various cancers. In hepatocellular carcinoma, HHLA2 is primarily expressed in the peritumor region, co-localizes with CD68+ macrophages and is associated with an immunosuppressive microenvironment, contributing to poor survival outcomes (Xu *et al.* 2021*b*, Wang *et al.* 2022). Interestingly, HHLA2 expression in kidney cancer cells is induced by TME signals *in vivo* but not *in vitro*, while monocytes can express HHLA2 in response to certain cytokines, particularly IL-10 (Shigemura *et al.* 2023). These findings underline the importance of the interaction between tumor cells and TME signals in the regulation of HHLA2. In addition, tumor heterogeneity may result in varied HHLA2 expression within different tumor regions, affecting interpretations, as observed for proteins of other immune checkpoint signaling pathways (Janakiram *et al.* 2015, Yang *et al.* 2022).

Two studies examined the expression of HHLA2 in the context of lung cancers, specifically focusing on its association with mutations in the epithelial growth factor receptor (EGFR) (Cheng *et al.* 2017, Chen *et al.* 2020). Both studies found that HHLA2 expression was significantly higher in tumors harboring mutations in this gene, suggesting a role for HHLA2 in modulating the immune microenvironment. This modulation may affect tumor progression and could serve as a potential biomarker for prognosis and inform treatment strategies in EGFR-mutated tumors. Although there are no direct studies on the mutational landscape and molecular profiling in endocrine-related tumors concerning HHLA2, the observed association between HHLA2 and specific mutations in other cancer types suggests that variants in regulatory or signaling genes in endocrine tumors might similarly affect HHLA2 expression. This hypothesis highlights the need for further research into how specific genetic and molecular characteristics of endocrine-related tumors could influence HHLA2 expression and the subsequent immunological behavior of these tumors.

In addition to these factors involving the TME and intrinsic tumor biomolecular context, discrepancies in findings regarding HHLA2 expression as a prognostic factor in endocrine-related tumors can be attributed to several other potential factors. First, differences in study populations, such as patient demographics (age and sex), cancer type and stage, can influence HHLA2 expression and its prognostic implications (Reitsema *et al.* 2020). For instance, older patients may exhibit higher HHLA2 expression, correlating with distinct clinical outcomes (Zhang *et al.* 2021). Second, the methodologies used for detection and analysis, including variations in immunohistochemistry techniques and evaluation criteria, can lead to differences in results (Leong & Leong 2011, Ding *et al.* 2022). The sensitivity and specificity of these methods, along with differing thresholds for classifying expression levels, can significantly impact findings (Leong 2004, Ding *et al.* 2022). Third, therapeutic contexts, including concurrent treatments such as chemotherapy or immunotherapy, can influence patient responses and modify the association between HHLA2 expression and prognosis. Although no specific studies exist for HHLA2 in this situation, patients with high HHLA2 expression may be more sensitive to chemotherapy and have better responses to immunotherapy, indicating that HHLA2 might play a role in modulating the tumor’s response to chemotherapeutic agents (Ding *et al.* 2022). Finally, study design and statistical analysis, including sample size and methods for adjusting confounding factors, and the tendency to analyze public repository data without conducting experimental validation, can lead to varied interpretations of HHLA2’s prognostic value. Understanding these factors is essential for comprehensively interpreting study outcomes and identifying the specific context of each research conclusion.

With few exceptions, endocrine malignancies are characterized by their slow proliferation rate, a trait that makes tyrosine kinase inhibitors effective for disease stabilization or, at best, achieving partial response (Sukrithan *et al.* 2023). Classical chemotherapies, which function by inducing DNA damage and halting cellular growth and division, have shown limited efficacy against these cancers. The gradual accumulation of mutations within cancer cells may enhance their ability to evade immune system detection, thus positioning these cells as suitable targets for immunotherapy (Kumar *et al.* 2021). Consequently, numerous clinical trials have investigated the use of anti-PD-LI therapy in the treatment of endocrine-related cancers (Latteyer *et al.* 2016).

HHLA2 exhibits broad expression in patients with PD-L1-negative cancers, making it a valuable target for immunotherapy alongside PD-L1. Targeting HHLA2 can benefit patients who are unresponsive to PD-1/PD-L1 inhibitors (Huang *et al.* 2022). For instance, gallbladder cancer, which typically shows a limited response to anti-PD-L1 therapy, often expresses higher levels of HHLA2 than PD-L1, suggesting that targeting HHLA2 could be a viable therapeutic strategy. Similarly, in clear cell renal cell carcinoma, low PD-L1 expression often limits the use of ICIs. These findings highlight the potential of HHLA2 as a novel target in cancer immunotherapy, with the possibility of enhancing the efficacy of ICIs. HHLA2 and PD-L1 can be concurrently targeted in patients who overexpress both checkpoints, which may include those with endocrine-related cancers (Huang *et al.* 2022, Wang *et al.* 2023). The development of bispecific antibodies targeting both PD-L1 and HHLA2 signaling has the potential to stimulate the immunogenic pathway and enhance responses to ICIs, making it a promising approach in cancer immunotherapy (Wang *et al.* 2019).

The exploration of HHLA2 within endocrine-related cancer research remains limited. The absence of a functional *HHLA2* gene in mice constraints animal studies, as most investigations have been conducted in humans (Zhao *et al.* 2013). To better understand HHLA2’s interactions with factors and signaling within the TME of solid cancers, more research using humanized tumor models is needed. A promising avenue for future studies involves investigating the efficacy of incorporating immune checkpoint genes into chimeric antigen receptor-modified T (CAR-T) cells for cancer immunotherapy. Among the alternative checkpoints explored thus far, HHLA2 has garnered increasing attention in this context. This strategy offers the advantage of directing HHLA2 CAR-T cells to the tumor site, thereby enhancing their anti-tumor efficacy. As no published work has assessed the impact of HHLA2 on CAR-T immunotherapy, it presents an intriguing focus for future research efforts (Majzner *et al.* 2019, Yang *et al.* 2020, Vitanza *et al.* 2023).

Despite these gaps, the existing body of the literature offers compelling preliminary evidence of HHLA2’s significance in endocrine-related cancers. This foundational groundwork encourages future investigations to employ sophisticated sequencing techniques and focus on precisely defined tumor subtypes. These efforts aim to elucidate HHLA2’s role and therapeutic potential in endocrine malignancies. Uncovering such insights may pave the way for innovative therapeutic interventions and improved patient care.

## Declaration of interest

The authors declare that no conflict of interest could be perceived as prejudicing the impartiality of the work reported.

## Funding

This work is supported by FAPESP (Fundação de Amparo à Pesquisa do Estado de São Paulo), Grant 2019/26780-9 and the 2023 AACR Maximizing Opportunity for New Advancements in Research in Cancer (MONARCA) Grant for Latin America, Grant number 23-15-01-BATI. The sponsors had no involvement in the study design, data collection, analysis, interpretation, report writing or the decision to submit the article for publication.
